# Fabrication of a Fluorocarbon Low Surface Energy Coating for Anti-Stain Applications

**DOI:** 10.3390/ma16247516

**Published:** 2023-12-05

**Authors:** Siwei Pan, Qing Hu, Yaohong Zhao, Qing Wang, Yuanyuan Li, Yihua Qian, Chunqing He

**Affiliations:** 1 Electric Power Research Institute of Guangdong Power Grid Co., Ltd., Guangzhou 510080, China; siweipan@163.com (S.P.); kennyaoo@126.com (Y.Z.); sdytwangqing@163.com (Q.W.); 13926035192@163.com (Y.Q.); 2 School of Physics and Technology, Wuhan University, Wuhan 430072, China; 2020202020067@whu.edu.cn (Q.H.); yanttely@whu.edu.cn (Y.L.)

**Keywords:** fluorocarbon resin, coating, SiO_2_, superhydrophobic, anti-stain performance

## Abstract

In the long-term working state, stains such as dust, oil, and charged particles in the environment are prone to deposit on the surface of the power equipment, which has great security risks. To achieve anti-stain performance, fluorocarbon composite coating with a low surface energy was prepared and studied. In this paper, SiO_2_ nanoparticles were used as inorganic fillers and fluorocarbon resin was used as the substrate to form anti-stain coatings. By adjusting and optimizing the ratio of fillers and organic resins, coatings with different static contact angles were constructed. The optimum composite coating has a contact angle of 151 ± 2° and a surface energy of 9.6 mJ/m^2^. After high-temperature treatment (up to 200 °C), immersion in corrosive solutions (pH 3–11), and sandpaper abrasion (after 5 abrasion cycles), the coating has been proven to show good thermal, chemical and mechanical stability. Our study provides significant research and market opportunities for the anti-stain application of the fluorocarbon composite coating on power equipment.

## 1. Introduction

With the development of urban switchgear, the scale and number of switchgear grew rapidly. Switchgear has been widely used in the construction of power distribution networks for its advantages of small size, convenient installation and easy maintenance [1]. Meanwhile, the insulating surfaces of power devices, for instance, high-voltage insulators and cables, are often subjected to harsh environmental conditions, including airborne pollutants and contaminants, which can compromise their functionality and lead to operational inefficiencies [2].

The development of superhydrophobic anti-stain coatings is one of the promising solutions [3,4]. Superhydrophobic surfaces are characterized by having a water contact angle greater than 150° and a roll-off angle less than 10°. These surfaces have been proven to have the potential to effectively mitigate stain problems owing to their adaptive characteristics. By minimizing the adhesion of contaminants and making them easy to remove, superhydrophobic coatings not only ensure the longevity of electrical equipment but also improve its operational reliability [5,6,7].

Superhydrophobic coatings typically feature two characteristics: a relatively low surface energy and hierarchical micro/nanostructures [8]. In general, such surfaces can be created by generating rough textures on low-surface-energy materials or by decorating surfaces with low-surface-energy compounds at the micro/nanoscale [9,10]. Currently, various approaches have been explored to fabricate superhydrophobic surfaces, including template methods, spray coating [11], self-assembly [12], sol-gel techniques [13], deposition method [14], etching processes [15] and electrochemical methods [16], all of which have been employed for fabricating superhydrophobic surfaces. Among these, spray coating is a convenient and economical fabrication method. It involves the use of low-surface-energy polymer organics and inorganic fillers as precursor sols or polymer solutions, which are then high-pressure sprayed onto the substrate surface. Once the solvent evaporates and dries, a coating is formed without the need for further processing, enabling the efficient production of hydrophobic coatings on a large scale. Spray coating offers the capability to control the surface roughness and hydrophobicity of coatings by altering the type, size, and content of inorganic fillers. Commonly used inorganic fillers include SiO_2_ [17]_,_ TiO_2_ [18] and Al_2_O_3_ [19] among others. Among these, nano-silica serves as a low-cost, biocompatible inorganic material with low surface energy. It can construct rough structures on the substrate’s surface, achieving excellent hydrophobic effects. In reports, it is generally functionalized by hydrophobic modifiers with low surface energy, for instance, fluorinated alkylsilanes (FAS) [20], hexadecyltrimethoxysilane (HDTMS) [21], and aminopropyltriethoxysilane (AP) [22] et al. Of these, fluorinated alkylsilanes are interesting in decreasing surface energy because of the specific characteristics of their fluorine atoms. It has also been reported that when surfaces are abundant in CF_3_ groups, fluorinated compounds can decrease the surface tension to ~6.7 mN/m, which is significantly lower than the surface tension of water (~72.8 mN/m at room temperature) [23]. Additionally, as the coatings are expected to endure in the complex operational environment of electrical equipment for an extended period, withstanding external forces and temperature variations, the prepared coatings must exhibit mechanical and thermal stability.

Despite the ability of spray coating technology to create micro-nano multilevel rough structures through continuous spraying and layer-by-layer deposition, the surface composite structure is fragile and susceptible to damage. To enhance adhesion to the substrate and cohesion among inorganic fillers, thus improving coating stability, it is necessary to combine it with organic resins. Recently, there have been reported attempts to use binding polymers including fluoroacrylic copolymers [24], polydimethylsiloxane (PDMS) [25], silicone resins [26] and epoxy resin [27]. Wu et al. [28] fabricated a polyester-silica coating by spray method, which displayed superhydrophobicity and anti-condensation properties at different subcooling temperatures. Liu et al. [29] prepared a superhydrophobic coating by incorporating low surface energy fluorine-silicone resin with poly (methyl-3,3,3-trifluoropropylsiloxane) (PMTFPS) modified silica. It is important to notice that the selection of the binding polymer is usually based on its surface energy, mechanical strength and adhesion properties, which significantly impacts the performance of the ultimate coating. Fluorocarbon resin is one of the most widely used resins [30,31]. It is produced by alternately copolymerising alkyl vinyl ether and vinyl trifluoride and consists primarily of C-C and C-F bond. The C-C primary bond is densely surrounded by fluorine atoms, forming a spiral structure, imparting fluorocarbon resin with a low surface energy of approximately 9–16 mJ/m^2^ [32], outstanding weather resistance, and serves as an excellent high and low-temperature insulation material [33,34,35].

In this paper, superhydrophobic surfaces were fabricated based on SiO_2_ nanoparticles and fluorocarbon resin using a facile spraying method. By adjusting the mass ratio of SiO_2_ to fluorocarbon resin, the optimum formulation coating is selected with a rough surface structure and low surface energy. The optimum formulation coating preparation process is simple, with excellent liquid repellence, good abrasion resistance and anti-stain performance, and is available for large-scale production. This study provides important research and application opportunities for fluorocarbon resin/SiO_2_ nanocomposite coating to achieve anti-stain properties.

## 2. Materials and Methods

### 2.1. Materials

Fluorocarbon resin (HLR) was purchased from Shanghai East Fluorine Chemical Technology Co., Ltd. (Shanghai, China). SiO_2_ nanoparticles (R-972) (20 nm in diameter) were purchased from Evonik Degusse Co., Ltd. (Beijing, China). Dodecafluoroheptylpropyltrimethoxysilane (G502) was purchased from Harbin Xuejia Fluorine Silicon Chemical Co., Ltd. (Harbin, China). Dispersant (BYK-104S) was acquired from BYK Chemie Company. (Wessel, Germany). The leveling agent (TEGO-300) was acquired from Germany TEGO Company. (Ludwigshafen, Germany). Propylene glycol methyl ether acetate (PMA) was bought from Jiangsu Hualun Chemical Co., Ltd. (Yangzhou, China). Butyl acetate was bought from Tianjin Bohuatong Chemical Products Sales Center. (Tianjin, China).

### 2.2. Methods

Firstly, 0.18 g nano-SiO_2_ particles and 0.2 mL G502 were dispersed in 2.5 mL butyl acetate. The SiO_2_ dispersion solution was obtained after 1 h ultrasonic dispersion. Then, 0.09 g fluorocarbon resin was ultrasonically dissolved in 400 mL PMA solvent to obtain a fluorocarbon resin solution. Finally, the SiO_2_ dispersion solution, 0.1 mL dispersant and 0.1 mL leveling agent were added to the fluorocarbon resin solution for ultrasonic dispersion for 30 min to obtain the fluorocarbon/SiO_2_ coating. At this additive amount, the mass ratio of SiO_2_ to fluorocarbon resin was 2:1. According to the above method, the coatings with a mass ratio of SiO_2_ to fluorocarbon resin of 1:3, 1:2, 1:1, 2:1, and 3:1 were prepared by varying the amounts of SiO_2_ and fluorocarbon resin without varying the amounts of other substances.

The glass slides (76.2 mm × 25.4 mm × 1.2 mm) were washed sequentially by ultrasonic shaking in appropriate amounts of acetone, DI water and anhydrous ethanol for 10 min to remove surface impurities, and then vacuum dried. A spray gun was used to spray on the surface of the slide. The distance between the nozzle and the glass slide was 15 cm. The spraying pressure was 0.5 MPa. The coated samples were placed in a fume hood for 12 h at room temperature to obtain five kinds of fluorocarbon/SiO_2_ composite coating samples with different surface structures.

### 2.3. Characterization

A field emission scanning electron microscope (FESEM, Hitachi Co., Ltd., S-4800, Tokyo, Japan) was employed to observe the surface morphology of the composite coatings. The voltage was 5 kV, the current was 2.25 A and the working distance was 9 mm. An atomic force microscope (AFM, Bruker, Karlsruhe, Germany) was applied to analyze the structure and roughness of the surface. The AFM scanning parameters were as follows: scanning frequency (0.2 Hz), peak force frequency (2 kHz), peak force amplitude (200 nm), and lift height (400 nm). Roughness parameters were obtained based on the AFM tests.

A contact angle measuring instrument (CA, Kino Industries Co., Ltd., SL200B, Boston, MA, USA) was used to characterize the droplet shape and contact angle. A droplet of 5 μL was applied for contact angle measurement. The contact angles were tested and recorded for deionized water, formamide, and diiodomethane. According to the Lifshitz-van der Waals/Lewis acid-base (LW-AB) method, the surface energy of the coating was calculated using the contact angles of the three fluids mentioned above. The average values of 3 positions were selected as the measurement results for each sample.

### 2.4. Thermal and Chemical Stability

The stability of the coatings was also assessed under harsh environmental conditions such as high temperatures, acidity, and alkalinity. For thermal stability tests, the coatings were heat-treated at 25 °C, 50 °C, 100 °C, 150 °C and 200 °C for 2 h, respectively. The chemical stability of the coatings was measured by soaking them in corrosive solutions (acidic or alkaline solution) at pH values of 3, 5, 7, 9, and 11 for 24 h. The corrosive solutions were formulated by diluting sulphuric acid or sodium hydroxide with DI water. The water contact angle was measured as the coating was returned to room temperature and dry.

### 2.5. Abrasion Persistence

The abrasion resistance of the coated samples was assessed by measuring the wetting behavior before and after abrasion (sandpaper). The coated side was moved downward on the 1000# sandpaper with the 50 g weight pressed on it. This procedure consists of a cycle of pushing it horizontally by 10 cm and then pushing it back into place in the opposite direction. The wettability of the coated surfaces was tested after multiple abrasions, and changes in the hydrophobicity of coatings were observed. Additionally, pictures and SEM images of superhydrophobic coatings were obtained after 5 abrasion cycles.

### 2.6. Anti-Stain Performance

The anti-stain ability of the coated surfaces was tested using Rhodamine B, milk, muddy water and cola as sources of pollutant. The coated glass slide was inclined at about 10°. Each of the above pollutants was dropped onto the coated glass slide from a 4 cm height. The movement of the droplet on the samples was recorded.

## 3. Results and Discussion

### 3.1. Surface Characteristics of Fluorocarbon Resin/SiO_2_ Nanocomposite Coatings

**Figure 1:**
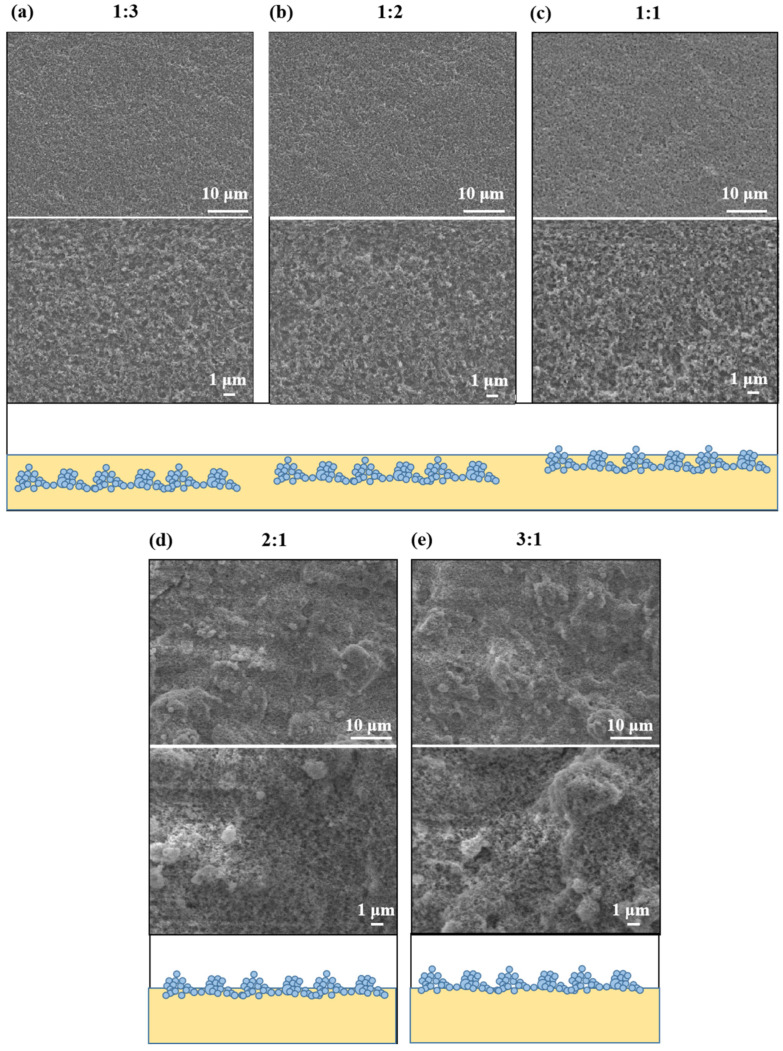
SEM images and schematic illustrations of fluorocarbon resin/SiO_2_ nanocomposite coatings with various mass ratios of SiO_2_ to fluorocarbon resin: (**a**) 1:3; (**b**) 1:2; (**c**) 1:1; (**d**) 2:1; (**e**) 3:1.

**Figure 2:**
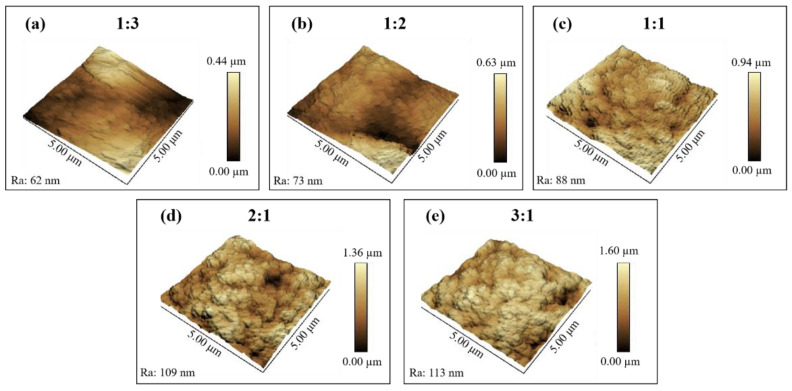
Three-dimensional AFM images of fluorocarbon resin/SiO_2_ nanocomposite coatings with various mass ratios of SiO_2_ to fluorocarbon resin: (**a**) 1:3; (**b**) 1:2; (**c**) 1:1; (**d**) 2:1; (**e**) 3:1.

The SEM images and schematic illustrations of nanocomposite coatings with different mass ratios of SiO_2_ to fluorocarbon resin (1:3, 1:2, 1:1, 2:1, 3:1) are as shown in Figure 1. The coatings were composed of SiO_2_ nanoparticles linked together by fluorocarbon resin, generating a rough surface. When a small number of SiO_2_ nanoparticles (1:3, 1:2, 1:1) is added, the coatings are only slightly rough, and the nanoparticles appear to be submerged in fluorocarbon resin (Figure 1a–c). As shown in Figure 1d,e and inset, at mass ratios of 2:1 and 3:1, SiO_2_ nanoparticles aggregate and form protrusions, resulting in micrometer-scale structural undulations on the surfaces of the coating. Fluorocarbon resin/SiO_2_ coatings have a particular micro-nanostructure, which is essential for improving the hydrophobicity of the coatings. In addition, the amount of SiO_2_ in the coating is a major influence on the formation of the micro-nanostructures required to attain superhydrophobicity. The micro-nano structure developed is a delicate balance between the resin and SiO_2_ nanoparticles. In this attractive surface structure, the rough structure is created by the silica nanoparticles and their aggregates and is sustained by the bonding of the resin. To further understand the morphology and roughness of the coatings, AFM experiments were also conducted, as shown in Figure 2. The coating (1:3) is slightly rough with a peak-to-valley distance of 0.44 µm (Figure 2a), whereas, as the mass ratio of SiO_2_ nanoparticles increases, the peak-to-valley distance shows 0.63 µm, 0.94 µm, 1.36 µm, 1.60 µm, respectively. The higher the SiO_2_ nanoparticle ratio, the higher the roughness of the coating, which is in agreement with the SEM results. Agglomerates of nanoparticles contribute to the formation of micron-sized features on the substrate. SiO_2_ nanoparticles cover the outer surface of the micron features and form nanoscale features. This multilevel micro-nanoscale surface structure is responsible for the increase in roughness. Moreover, the AFM results show that the coatings (2:1, 3:1) have peak-to-valley distance (1.36 µm, 1.60 µm) and roughness (Ra) (109 nm, 113 nm). The surface of the coating reaches the micrometer scale in view of the height change and has significant structural fluctuations at the nanometer scale. This finding also demonstrates that the fluorocarbon resin/SiO_2_ coating surface features a micro-nanostructure.

**Figure 3:**
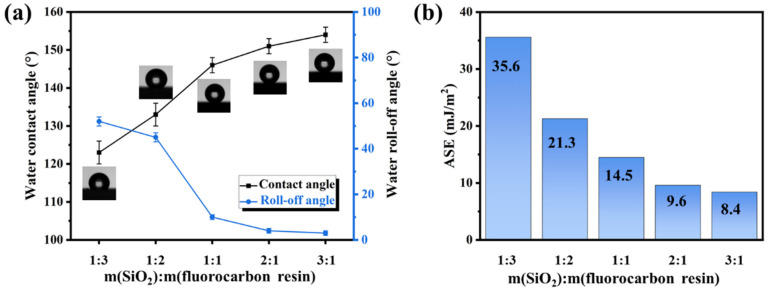
(**a**) The wettability and (**b**) ASE of fluorocarbon resin/SiO_2_ nanocomposite coatings with various mass ratios of SiO_2_ to fluorocarbon resin.

The influence of the mass ratio of SiO_2_ to fluorocarbon resin on wettability was investigated, and the water roll-off and contact angles were measured (Figure 3a). The coating (1:3) displays a roll-off angle of 52 ± 2°and a water contact angle of 123 ± 3°. With an increase in the mass ratio of SiO_2_ to fluorocarbon resin, the roll-off angle reduces, and the water contact angle of the coatings increases. The superhydrophobicity was obtained with a water roll-off angle of less than 5° and contact angle of more than 150° when the mass ratio was 2:1 and 3:1. The coating (3:1) possesses exceptional hydrophobicity with a water roll-off angle of 3 ± 1° and contact angle of 154 ± 2°. However, as mentioned in Section 3.2, the abrasion resistance of the coating is significantly diminished at this mass ratio. In addition to water, the contact angles of further liquids (diiodomethane, formamide) were also recorded and analyzed by the LW-AB method. As shown in Figure 3b, the apparent surface energies (ASE) of the coatings with 1:3, 1:2, 1:1, 2:1, and 3:1 mass ratios of SiO_2_ to fluorocarbon resin are 35.6, 21.3, 14.5, 9.6, and 8.4 mJ/m^2^, respectively. The outstanding liquid repellency of the superhydrophobic surfaces is essentially attributed to their surface roughness and low surface energy [36].

Table 1 lists the roughness parameters of nanocomposite coatings with different mass ratios, where the root-mean-square roughness (Rq), the mean roughness (Ra), and the height span (z) are the AFM characterization results and the area fraction of the liquid-air contact $f_{lv}$ is derived from the Cassie-Baxter relation [37] of the Cassie model. It can be found that the Rq, Ra and z of the composite coatings gradually become higher with the growth of the SiO_2_ ratio, which coincides with the change in the roughness of the coatings surface in the SEM image. As the surface structural roughness grows, the water contact angle of the sample also increases. 

The above experimental results can be interpreted from the Cassie-Baxter relation:

(1) $$cos\theta =\left({1-f}_{lv}\right)(cos\theta_{e}+1)-1$$

where $\theta_{e}$ is the intrinsic contact angle between the liquid and the smooth solid surface ($\theta_{e}$ = 94°). As can be seen from Table 1, the contact area between the liquid and air ($f_{lv}$) gradually increases with the addition of the SiO_2_ mass ratio, reaching 89% for the composite coating (3:1). When the amount of SiO_2_ is insufficient, it is submerged in the fluorocarbon resin. The surface is relatively smooth so that there is less air layer on the surface of the coating. However, enough SiO_2_ nanoparticles form protrusions, which increases the surface roughness, which makes droplets exist in the state of Cassie to achieve superhydrophobicity properties [38]. Therefore, the trend is consistent between the air layer on the surface of composite coatings with different mass ratios and their actual surface roughness.

### 3.2. Stability of Fluorocarbon Resin/SiO_2_ Nanocomposite Superhydrophobic Coatings

**Figure 4:**
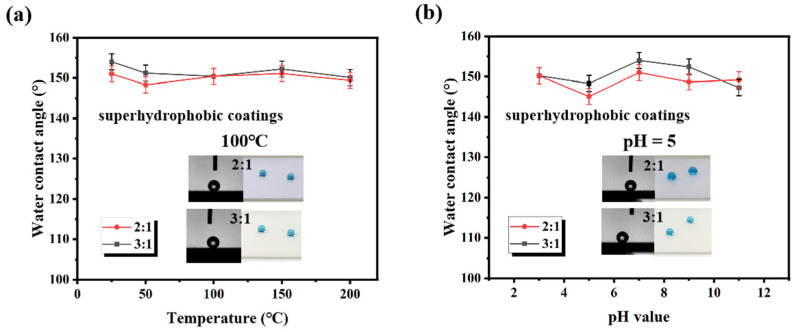
The contact angle of the superhydrophobic coatings (The mass ratio of SiO_2_ to resin is 2:1 and 3:1) (**a**) after 2 h treatment at different temperatures and (**b**) in different pH test solutions.

High-temperature and acidic, alkaline conditions exist in some power applications. Hence, it is critical to assess the ability of the prepared superhydrophobic surfaces to withstand these harsh environments. Figure 4a reveals the resistance of superhydrophobic coatings (The mass ratio of SiO_2_ to fluorocarbon resin is 2:1 and 3:1) to high temperatures. Before the water contact angle test, the coatings were heat-treated at 25 °C, 50 °C, 100 °C, 150 °C and 200 °C for 2 h. As the samples were returned to room temperature, the water contact angle was measured. It can be seen that the fluctuations in contact angle values are faint, which indicates that the coatings can maintain their excellent hydrophobicity against high-temperature environments. This is due to the advantages of the chemical structure of fluorocarbon resin compared to conventional resins. The bond energy of C-F (485 kJ/mol) is significantly larger than that of C-C (332 kJ/mol) and C-O (326 kJ/mol), which makes fluorocarbon resins more resistant to high temperatures [39]. Moreover, the maximum temperature tested (200 °C) was not sufficient to cause significant softening of the fluorocarbon resin and thus deformation of the microstructure. Figure 4b shows the water contact angle tests of the samples after 24 h of immersion in an acidic or alkaline environment, respectively. Even under corrosive conditions, the samples still keep excellent water repellency. The good resistance to a corrosive environment is attributed to the chemical stability of the nanocomposite constituents (fluorocarbon resin and SiO_2_) because it is difficult to be destroyed by acidic or alkaline solutions. For the superhydrophobic coating, it is essential for applications in harsh outdoor environments.

**Figure 5:**
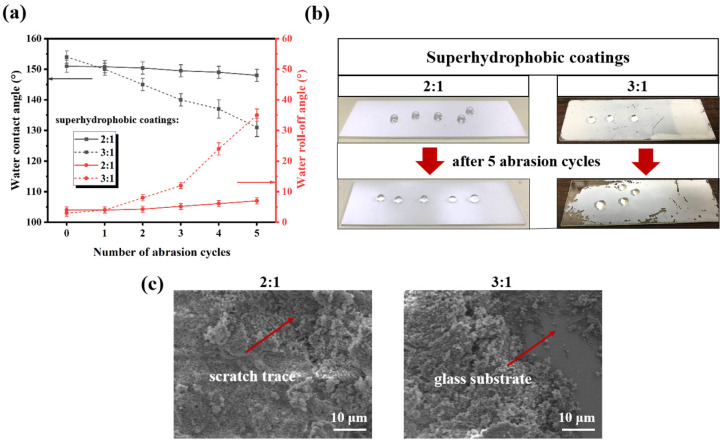
(**a**) The water wettability of superhydrophobic coatings (The mass ratio of SiO_2_ to fluorocarbon resin is 2:1 and 3:1) after abrasion resistance test; (**b**) Pictures of superhydrophobic coatings after 5 abrasion cycles; (**c**) SEM images of superhydrophobic coatings after 5 abrasion cycles.

It is well known that abrasion resistance is essential to the widespread application of superhydrophobic materials. After friction, the wettability of the coatings was assessed by water contact and roll-off angle measurements, as shown in Figure 5a. When the superhydrophobic coating friction 0, 1, 2, 3, 4, 5 cycles, the water contact angle of superhydrophobic coating (3:1) decreases from 154 ± 2° to 131 ± 3°, but that of the superhydrophobic coating (2:1) decreases from 151 ± 2° to 148 ± 2°. Correspondingly, the water roll angle of the former increases from 3 ± 1° to 35 ± 2°, while that of the latter increases only from 4 ± 1° to 7 ± 1°. By comparison, we can conclude that the abrasion resistance of the nanocomposite superhydrophobic coating (2:1) is much better than that of the superhydrophobic coating (3:1). When the mass ratio of SiO_2_ to resin is 3:1, a portion of the glass substrate is observed to be exposed after five abrasion cycles (Figure 5b,c). This is because the quantity of fluorocarbon resin used as a binding polymer in the coating is not sufficient to maintain the stability of the surface structure. Owing to the lack of fluorocarbon resins, many SiO_2_ nanoparticles are unable to form stable roughness microfeatures and instead occupy the spaces between the microfeatures [28], as confirmed by the SEM (Figure 1e). Comparatively, when the mass ratio of SiO_2_ to resin is 2:1, the superhydrophobic coating has less coating shedding, only a slight powder phenomenon, and still maintains strong hydrophobic properties (148 ± 2°) after 5 abrasion cycles. Despite the visible scratch traces, the rough surface with micro-nanostructures is maintained (Figure 5c). This result is due to the adhesive reinforcement of the fluorocarbon resin, which holds the SiO_2_ nanoparticles and their aggregates firmly on the surface, thus maintaining the stability of the surface’s rough structure. Therefore, controlling the proportion between the inorganic filler and the resin has a significant impact on the abrasion resistance and hydrophobicity of the coating. At the appropriate ratio (2:1), Under the action of resin bonding SiO_2_ nanoparticles can form a stable rough structure, which gives them excellent abrasion resistance and superhydrophobic properties.

Most superhydrophobics are limited in practical application due to their poor stability. In this research, since the stability of the coatings with various mass ratios is consistent under high temperatures and acid-base conditions, it was determined that this mass ratio (2:1) of coatings provided the best compromise between superhydrophobicity and abrasion resistance. We describe a scalable approach to fabricating optimum formulation superhydrophobic coatings (2:1) with thermal stability, acid and alkali resistance, and abrasion resistance.

### 3.3. Anti-Stain Performance of Optimum Formulation Superhydrophobic Coating

**Figure 6:**
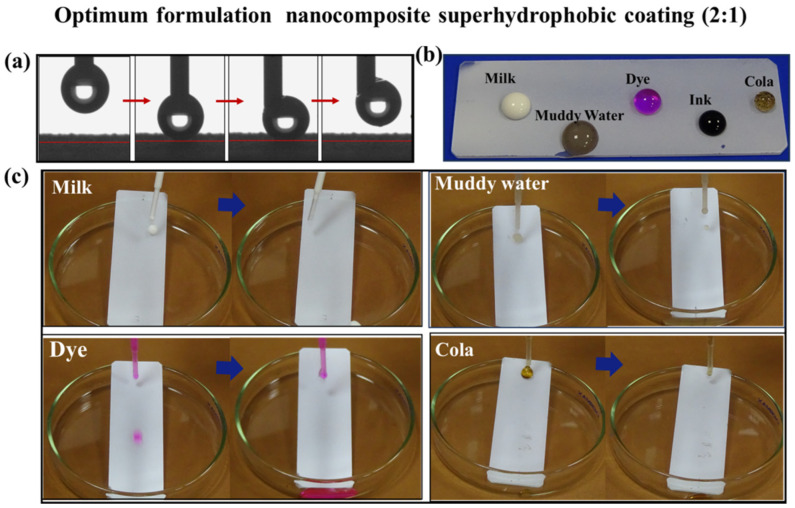
Anti-stain performance of optimum formulation superhydrophobic coating: (**a**) dynamic characteristics of water droplets on the coating; (**b**) Wettability of different liquids on the coating; (**c**) photos of different liquids rolling off the coating.

Figure 6 shows the anti-stain performance of optimum formulation superhydrophobic coatings (2:1). To prove the anti-stain performance of the optimum coating, Rhodamine B, milk, muddy water and cola were, respectively, used as stain sources to test the coating surface, and the experimental results were shown in Figure 6c. When the pollutant droplets fell onto the coating surface, they were first bounced and then rolled along the surface. There were almost no residual pollutants on the surface of the optimum coating, which displays excellent anti-stain performance. The anti-stain performance of the coating is primarily attributed to its low surface energy and the existence of a large amount of air in the rough structures. The presence of the air layer decreases the contact area between the solid and the liquid when a droplet hits the surface, effectively reducing the adhesion between the pollutant and the sample surface [36]. This behavior is confirmed through the dynamic properties of water drops in contact with the superhydrophobic coating (Figure 6a), which shows that the adhesion of the coating to the water drops is low. Firstly, a drop of water was deposited on the surface of the coating with a needle and then pushed down so that the drop was visibly deformed. Then, the needle was lifted, and the water droplet was lifted with the needle rather than staying on the surface of the coating. This result demonstrates that water drops are not easily adhered to the coating surface. Moreover, Figure 6b displays the various liquids on the coated surface. All the droplets on the coated surface are spherical, indicating that the coating keeps excellent repellency for the different liquids.

**Figure 7:**
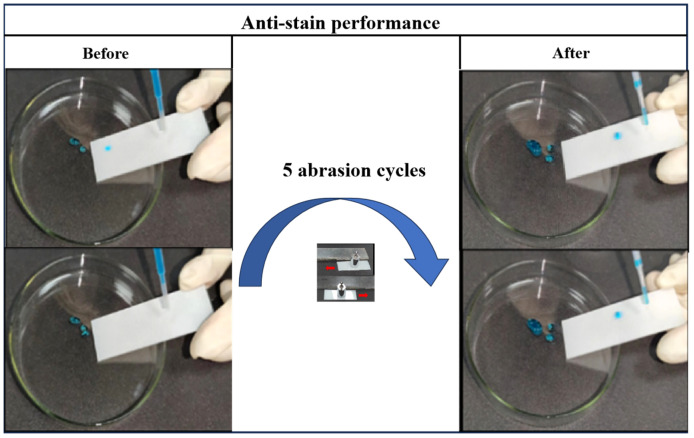
Anti-stain performance of optimum formulation superhydrophobic coating after 5 abrasion cycles.

It can be observed that after 5 abrasion cycles, the water droplets still rolled off the surface of the coating easily in Figure 7. This result indicates that optimum formulation superhydrophobic coatings possess excellent anti-stain performance. The stain resistance of the coating can reduce the accumulation of stains, thereby lowering the risk of damage to the surface of the power equipment, and conserving resources for cleaning up the stains.

## 4. Conclusions

In summary, superhydrophobic fluorocarbon/SiO_2_ composite coating was fabricated by using the spraying method for anti-stain applications. When SiO_2_ nanoparticles were incorporated with fluorocarbon resins with low surface energy, the resultant superhydrophobic properties were dependent on the surface roughness structure and low surface free energy. It was obtained by optimal balance between the resin and the SiO_2_ nanoparticles. At lower mass ratios, the majority of the nanoparticles were embedded in the resin and therefore had no significant improvement in hydrophobic properties. However, superhydrophobicity was attained when the mass ratio of SiO_2_ to fluorocarbon resin was 2:1, due to the formation of a steady rough surface structure. Additionally, it was also found that the higher the mass ratio (3:1), the poorer the bond strength, leading to significant removal of the coating from the substrate. The optimum formulation coating (2:1) exhibited a high contact angle (151 ± 2°), a low roll-off angle (4 ± 1°) and low surface free energy (9.6 mJ/m^2^). The coating showed excellent hydrophobicity even after high-temperature treatment and immersion in corrosive solutions, demonstrating good thermal and chemical stability. Even after 5 abrasion cycles, the coating still displayed good mechanical stability of the coating. This nanocomposite exhibits excellent properties, thereby proving to be a prospective candidate for facile production and large-scale economic of superhydrophobic surfaces subjected to harsh environments.

## Figures and Tables

**Table 1:** The roughness parameters of samples with various mass ratios.

Sample/m(SiO_2_):m(Resin)	R_q_/nm	R_a_/nm	z/µm	θ/°	$f_{lv}$/%
1:3	73	62	0.44	123 ± 3	51
1:2	86	73	0.63	133 ± 3	66
1:1	106	88	0.94	146 ± 2	82
2:1	121	109	1.36	151 ± 2	87
3:1	135	113	1.60	154 ± 2	89

## Data Availability

Raw data are available upon request.
